# Application of stereotactic body radiotherapy in advanced pancreatic cancers in Australia

**DOI:** 10.1002/jmrs.313

**Published:** 2018-11-09

**Authors:** Laurence Kim, Nam Nguyen, Nimit Singhal, Vinh‐An Phan, Ivan Iankov, Hien Le

**Affiliations:** ^1^ Department of Radiation Oncology Royal Adelaide Hospital Adelaide South Australia Australia; ^2^ Department of Gastroenterology Royal Adelaide Hospital Adelaide South Australia Australia; ^3^ Department of Medical Oncology Royal Adelaide Hospital Adelaide South Australia Australia; ^4^ School of Medicine University of Adelaide Adelaide South Australia Australia

**Keywords:** Locally advanced, pancreatic cancer, SBRT, stereotactic body radiotherapy, unresectable

## Abstract

**Introduction:**

The majority of pancreatic cancers present locally advanced and carry a high mortality rate. Treatment is challenging, with mixed data suggesting use of chemotherapy alone or in combination with radiotherapy. The use of radiotherapy has previously been limited due to lack of ability to deliver radiation to the tumour mass without causing significant toxicity to surrounding organs. Stereotactic body radiotherapy (SBRT) allows delivery of higher biologically equivalent dose in a shorter treatment duration. We sought to investigate the safety and application of this technique in our centre.

**Method:**

We enrolled 27 patients from 2015, identified as locally advanced unresectable with histologically confirmed, non‐metastatic, pancreatic adenocarcinoma. All patients had endoscopically inserted fiducial markers and where possible concurrent chemotherapy was administered. Dose schedules ranged from 25 to 42 Gy in 5 or 3 fractions.

**Results:**

With an overall median follow up of 9 months (range, 3–32.7), the median survival was 11.6 months. Of those alive at 1 year, the local control rate was 67%. Six patients had Grade 3 toxicity, and other six had Grade 2 toxicity. None had Grade 4 or above toxicity. The most common symptom recorded was fatigue.

**Conclusion:**

SBRT for locally advanced pancreatic cancer is technically complex but feasible in a high volume centre. SBRT is unique, allowing safe delivery of high radiation dose resulting in good local control and decreases treatment time making it an attractive option for patients with unresectable pancreatic cancer.

## Introduction

Pancreatic cancer remains one of the most aggressive malignancies and is the fifth most common cause of cancer‐related deaths in Australia.^1^ The number of new cases in Australia increased from 1206 in 1982 to 2865 in 2013 and is predicted that by 2030, pancreatic cancer will be the second highest cause of cancer mortality. The overall 5 years survival rate for pancreatic cancer is less than 8%, and this rate has not significantly improved in the past 30 years.^1^ Complete surgical resection remains the only means of providing long‐term control and potential cure.^2^ However, a majority of patients present late in their disease with up to 85% being unresectable, either from metastatic disease or locally advanced disease, leading to even worse overall survival.^3^

Currently, the standard treatment for these patients involves chemotherapy alone with conflicting results for the addition of radiation treatment. A more recent trial, LAP07, failed to demonstrate a statistically significant difference in the median survival between chemoradiotherapy compared to chemotherapy alone. However, it showed improvement in local control and the potential quality of life afforded by the addition of radiotherapy through delayed or decreased need for salvage therapy.^4^ In addition, Iacobuzio‐Donahue et al.^5^ demonstrated that up to 30% died from locally obstructive disease with few or no distant metastases. This highlights the potential benefit and importance of local therapy in the management of locally advanced pancreatic cancer.

The use of radiotherapy has been limited in the past due to the lack of ability to deliver the radiation to the pancreatic mass without causing significant toxicity to surrounding organs. Conventional radiotherapy requires approximately 6 weeks of daily treatment, which is significant for what is otherwise a disease with poor prognosis. Stereotactic body radiotherapy (SBRT) is a minimally invasive, innovative treatment option that allows delivery of a higher biologically equivalent dose, in a shorter treatment duration with minimal side effects. We investigated the application and safety of SBRT for locally advance unresectable pancreatic cancer with the use of endoscopic ultrasound (EUS) inserted fiducials and concurrent chemotherapy.

## Methods

### Patients and eligibility

This was a pilot prospective feasibility trial and we have data from 27 consecutively treated patients with biopsy proven non‐metastatic, locally advanced, unresectable pancreatic cancer at the Royal Adelaide Hospital between July 2015 and December 2017. The patients were deemed inoperable from assessment of a multi‐disciplinary team. Typical criteria include solid tumour contact with superior mesenteric artery (SMA) or celiac artery by more than 180°, celiac plexus involvement or unreconstructible superior mesenteric vein (SMV). Patients were included if they were 18 years or older, had Eastern Cooperative Oncology Group (ECOG) performance score of 0–2, with projected life expectancy of at least 6 weeks. Exclusion criteria included prior abdominal radiotherapy, other serious uncontrolled medical conditions or ECOG performance scores of 3 or 4. The study was approved by our institutional ethics committee and before receiving treatment, all patients provided written informed consent.

### Treatment planning

Three to four gold fiducial seeds were inserted using preloaded EUS needles outlining the tumour periphery. Computed tomography (CT) planning images were obtained with intravenous contrast, 3 mm slices and multi‐detector 4DCT. The patient underwent imaging and radiotherapy in the supine position, with arms up and full body immobilisation using the OmniV SBRT system (Bionix Radiation Therapy, Toledo, OH, USA). Treatment planning utilised fusion of magnetic resonance imaging (MRI) and/or positron emission tomography (PET) scan to ensure accurate delineation of the target volume. Free breathing technique was used with utilisation of 4DCT and internal target volume (ITV) concept to account for all phases of breathing. The gross tumour volume (GTV) was defined as the tumour based on the fused MRI and/or PET scan. The planning target volume 1 (PTV1) included an expanded uniform 5 mm margin. The planning target volume 2 (PTV2) was a smaller volume where a copy of PTV1 was made, but edited to include area of vessel abutment and boosted to a higher dose.

### Radiation dose and delivery

Our initial PTV1 dose prescription was 25 Gy in 5 fractions which was later adapted to 30 Gy in 5 fractions in our protocol. PTV2 was prescribed to 35 Gy and delivered as a simultaneous integrated boost. The prescription isodose encompassed at least 95% of the PTV. Dose constraints for the organs at risk followed the Timmerman guidelines.^6^ Total treatment was 5 fractions delivered at least 48 h apart. Daily on‐board imaging was utilised with cone‐beam CT matching to fiducials with 0 mm tolerance for couch shifts. Treatment was delivered using flattening filter free linac based volumetric modulated arc radiotherapy (VMAT) technique. Majority of patients received 3 months of neoadjuvant chemotherapy involving Fluorouracil, Leucovorin, Irinotecan and Oxaliplatin (FOLFIRINOX), then concurrent Capecitabine during SBRT.

### Follow‐up

The patients were examined 1 month after SBRT and every 3 months thereafter by the treating radiation oncologists. At each follow up, clinical examination, CA 19‐9, CT scan and toxicity profile were collected. Local control was defined by stable findings on CT scan with absence of local, nodal or distant progression. Acute toxicity was defined as adverse events occurring less than 3 months after SBRT and long term was defined as those occurring after 3 months. Toxicity was graded using the Common Terminology Criteria for Adverse Events v4.03. The local control and length of survival were calculated from the initiation of SBRT to progression or death.

### Statistical analysis

Statistical analysis was carried out using R statistical software version 3.4.2 for Windows. The comparisons between arms and subgroups were performed using Chi‐square and Fisher's exact test for categorical response variable and *t*‐test for quantitative response variables. Univariate analysis for survival was performed using the Kaplan‐Meier method and differences in Kaplan‐Meier curves were tested for statistical significance using the log rank test. Cox regression analysis was carried out to estimate the effect of significant factors on overall survival rate. The optimal Cox regression model was developed using a log‐likelihood ratio statistical test to compare several candidate models, including those considering interactions. Once the optimal statistical model was selected, it was used to produce adjusted survival curves for various subgroups of patients.

## Results

Table 1 lists the patient and treatment characteristics. Of the 27 patients, 10 received 25 Gy in 5 fractions with 35 Gy simultaneous integrated boost to the area of vessel abutment. The initial dose of 25 Gy was used in our protocol to assess the safety and feasibility and once we were confident in treatment planning and delivery, our protocol was developed into 30 Gy with 35 Gy simultaneous integrated boost. All but five patients received concurrent chemotherapy with oral Capecitabine. Three patients were deemed unfit for chemotherapy due to their age (>80 years old) or with significant medical comorbidities. The radiation dose was escalated to 36 Gy in 3 fractions for patients who did not have chemotherapy. One patient had protocol deviation where a higher dose of 42 Gy in 3 fractions was delivered. This patient in particular was young and fit with an isolated nodal recurrence following Whipple's procedure and adjuvant chemotherapy. This was feasible as the volume was small and away from organs at risk, allowing safe dose escalation.

**Table 1:** Baseline characteristics (*n* = 27)

	*n*	%
Median age (range)	74 (56–92)	
ECOG		
0	2	7
1	20	74
2	5	19
Stage		
IB	2	7
IIA	11	41
IIB	2	7
III	11	41
Recurrent	1	4
Median size (cm, range)	3.3 (1.5–6.3)	
Median initial CA 19‐9 (U/mL, range)	173 (2–6779)	
Concurrent chemotherapy		
Yes	22	81
No	5	19
Radiation dose		
25 Gy/5 F, 35 Gy SIB	10	37
30 Gy/5 F, 35 Gy SIB	13	48
36 Gy/3 F	3	11
42 Gy/3 F	1	4

F, fractions; SIB, simultaneous integrated boost.

**Figure 1:**
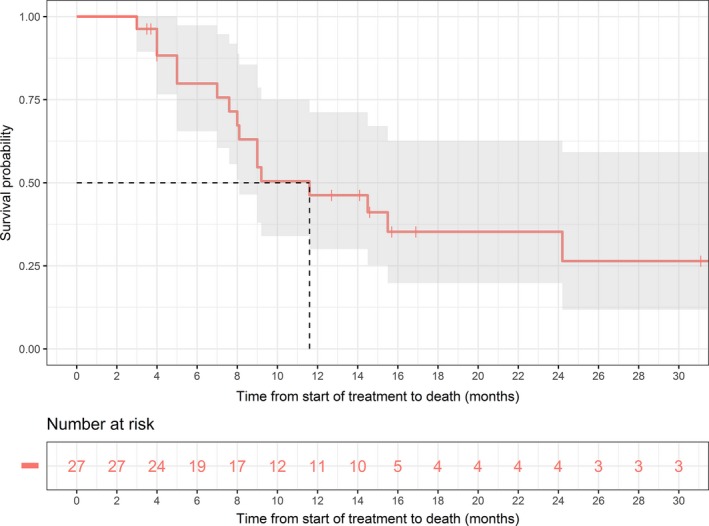
Kaplan‐Meier curve of overall survival.

**Figure 2:**
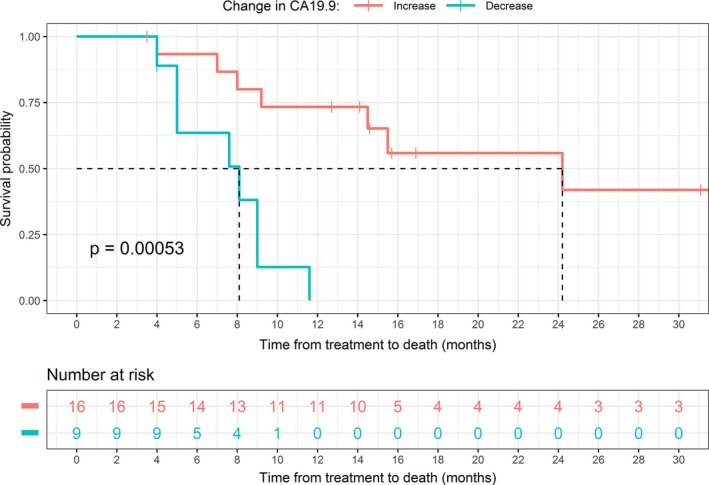
Kaplan‐Meier curve of survival with increase and decrease in CA 19‐9 with survival.

At median follow up of 9 months (range 3–32.7 months), the median length of overall survival was 11.6 months (Fig. 1). Of the cohort of patients alive at 1 year, the local control rate was 67%. The median CA 19‐9 was 173 U/mL. We had two missing data on the post‐treatment CA 19‐9 values which had to be excluded in our CA 19‐9 analysis. Figure 2 demonstrates the relationship between survival and change in CA 19‐9. As expected, a decrease in CA 19‐9 post‐treatment was associated with significant longer survival with a *P* value of 0.00053. Of the 27 patients, six were known to have developed distant metastases and 13 achieved initial regression of their local disease (Table 2).

**Table 2:** Results

1 year local control	67%
Initial radiological	
Regression	13 (48%)
Stable	6 (22%)
Progression	8 (30%)
Progression	
Metastatic disease	6 (22%)
Overall survival	11.6 months

PFS, progression free survival.

All of our patients had Grade 3 or less acute toxicity with majority having Grade 1 toxicity (33%). Six patients (22%) each experienced Grade 2 and Grade 3 acutetoxicity. These included fatigue, nausea and abdominal pain. Five of the six patients with Grade 3 toxicity received a dose of 25 Gy in 5 fractions with 35 Gy simultaneous integrated boost. The one other patient with grade 3 toxicity received a higher dose without chemotherapy of 36 Gy in 3 fractions. Another six patients experienced no toxicity at all (Table 3). Fatigue was the most common side effect experienced by our cohort, occurring in 13 patients (Table 4). Chemotherapy associated toxicities like diarrhoea and skin toxicities were not seen.

**Table 3:** Toxicity grade (CTCAE v4.03)

	No. (%)
Grade 0	6 (22)
Grade 1	9 (33)
Grade 2	6 (22)
Grade 3	6 (22)
Grade 4	0 (0)

CTCAE, common terminology criteria for adverse events; No, number.

**Table 4:** Common toxicity type

	No. (%)
Fatigue	13 (48)
Nausea/vomiting	9 (33)
Pain	6 (22)
Decreased appetite	5 (19)

No, number.

## Discussion

The technological development in the field of radiation, in particular SBRT, has made delivery of radiation extremely precise allowing sparing of normal organs more efficiently, whilst simultaneously delivering a higher biological dose over a shorter treatment time, thereby improving the therapeutic window.

This is particularly desirable in the treatment of locally advanced pancreatic cancers where obtaining durable local control is important and beneficial, but at the same time, being confronted by the intimate position of the tumour in relation to radiosensitive gastroduodenal structures.

To date, the role of radiotherapy in the treatment paradigm for patients with locally advanced pancreatic cancer has been conflicting. Older trials, including the Gastrointestinal Tumour Study Group (GITSG) 9283 and Eastern Cooperative Oncology Group (ECOG) 4201 demonstrated improved survival with chemoradiotherapy compared to chemotherapy alone. However, this also resulted in significant increased toxicity.^7^, ^8^ In contrast, the French study published in 2008 demonstrated decreased overall survival rates undergoing combined chemoradiotherapy compared to gemcitabine alone.^9^ With the aim to further clarify the uncertainty regarding the role of standard chemoradiotherapy, the phase III LAP07 trial was developed. This evaluated the role of chemoradiotherapy following induction chemotherapy compared to chemotherapy alone. The investigators reported no significant improvement in overall survival. However, the addition of radiation therapy was associated with decreased local progression. Even with this improvement, local control is poor which is consistent with most studies involving conventional three‐dimensional conformal radiation therapy. Importantly, poor local control often causes pain and/or obstructive symptoms that negatively affect patient's quality of life. Iacobuzio‐Donahue et al.^5^ demonstrated up to 30% of pancreatic cancer patients died from locally obstructive disease with few or no distant metastases. These findings highlight the potential benefit and importance of local radiation therapy in the management of locally advanced pancreatic cancer and also the need for further advancement in technique to improve local control.

Non‐randomised evidence investigating SBRT in pancreatic cancer has demonstrated very encouraging results with excellent locoregional control rates when compared to standard treatment paradigms. Reported 1 year locoregional control rates ranged from 50% to 94% (Table 5) compared to the 54% local control rate using conventional radiotherapy technique reported by Hammel et al.^4^, ^10^, ^11^, ^12^, ^13^, ^14^, ^15^, ^16^, ^17^, ^18^, ^19^, ^20^ Pooled analysis of 18 SBRT studies published in 2017 reported median overall survival of 17 months (range 5.7–47 months), superior to current patient outcomes with conventional treatments.^23^ Our cohort's median overall survival of 11.6 months, and 1 year local control of 67% were within the range of the other SBRT studies outside of Australia.

**Table 5:** Literature summary in pancreatic SBRT

References	Patients	Dose	1 year local control (%)	MS (months)	Chemo
Koong et al^10^	16 LA	25 Gy × 1	94	8.3	5‐FU concurrent
Didolkar et al^11^	85 LA/LR	5–10 Gy × 3	92	18.6	Post‐SBRT GEM
Polistina et al^12^	23 LA	10 Gy × 3	50	10.6	6 weeks induction GEM
Mahadevan et al^13^	39 LA	8–12 Gy × 3	85	20	2c induction GEM
Schellenberg et al^14^	20 LA	25 Gy × 1	94	11.8	1c induction + post‐SBRT GEM
Goyal et al^15^	19 LA/LR	20–25 Gy × 1, 8–10 Gy × 3	81	14.4	5‐FU or GEM
Lominska et al^16^	28 LA/LR	4–8 Gy × 3–5	86	5.9	5‐FU or GEM prior to SBRT
Chuong et al^17^	73 BR/LA	5–10 Gy × 5	81	16.4 BR, 15 LA	3 cycles GTX
Gurka et al^18^	38 BR/LA	5–6 Gy × 5	79	12.3	Post/concurrent‐ GEM or 5‐FU
Moningi et al^19^	88 BR/LA	5–6.6 Gy × 5	61	18.4	Peri‐SBRT GEM or 5‐FU
Herman et al^20^	49 LA	6.5 Gy × 5	78	13.9	Pre‐SBRT GEM
Lin et al^21^	20 LA	7–9 Gy × 5	70	20	Concurrent GEM
Tozzi et al^22^	30 LA/LR	7.5 Gy × 6	77	11	Pre‐SBRT GEM based

LA, locally advanced; 5‐FU, 5‐fluorouracil; LR, locally recurrent; SBRT, stereotactic body radiotherapy; GEM, gemcitabine; MS, median survival; BR, borderline resectable; GTX, gemcitabine, taxotere, xeloda; c, cycle.

Furthermore, SBRT is given over a limited number of fractions substantially decreasing the overall treatment time compared to the conventional 5–6 weeks chemoradiotherapy course. This allows fewer interruptions in systemic therapy, which may ultimately improve outcome based on the high rates of distant metastasis seen within this patient population. Additionally, given the poor prognosis of these patients, life expectancy is very short. SBRT is able to minimise patient's time spent undergoing treatment, thereby potentially improving their quality of life.

SBRT regimens have been associated with less incidence of Grade 3 and above acute toxicities compared with conventionally fractionated radiation therapy.^24^, ^25^, ^26^ However, late gastroduodenal complications with ulcers and bleeding have been seen in earlier studies, especially those with single fraction SBRT at a rate of 10–47%.^11^, ^27^, ^28^ More recent studies using hypofractionated SBRT (3–5 fractions) have shown reduced rates of late gastroduodenal toxicity compared to single fractionated treatment, hence our reasoning behind a 5 fraction regimen.^17^, ^20^, ^29^ Investigators from South Korea have also reported that patients treated over consecutive days experienced higher rates of toxicity suggesting that increasing interfraction interval to greater than 24 h reduced side effects.^30^ The prescription used in our protocol was 30 Gy in 5 fractions over 2 weeks with a simultaneous integrated boost of 35 Gy to area of vessel abutment. This equated to BED10 of 48 and 59.5 Gy respectively.

**Figure 3:**
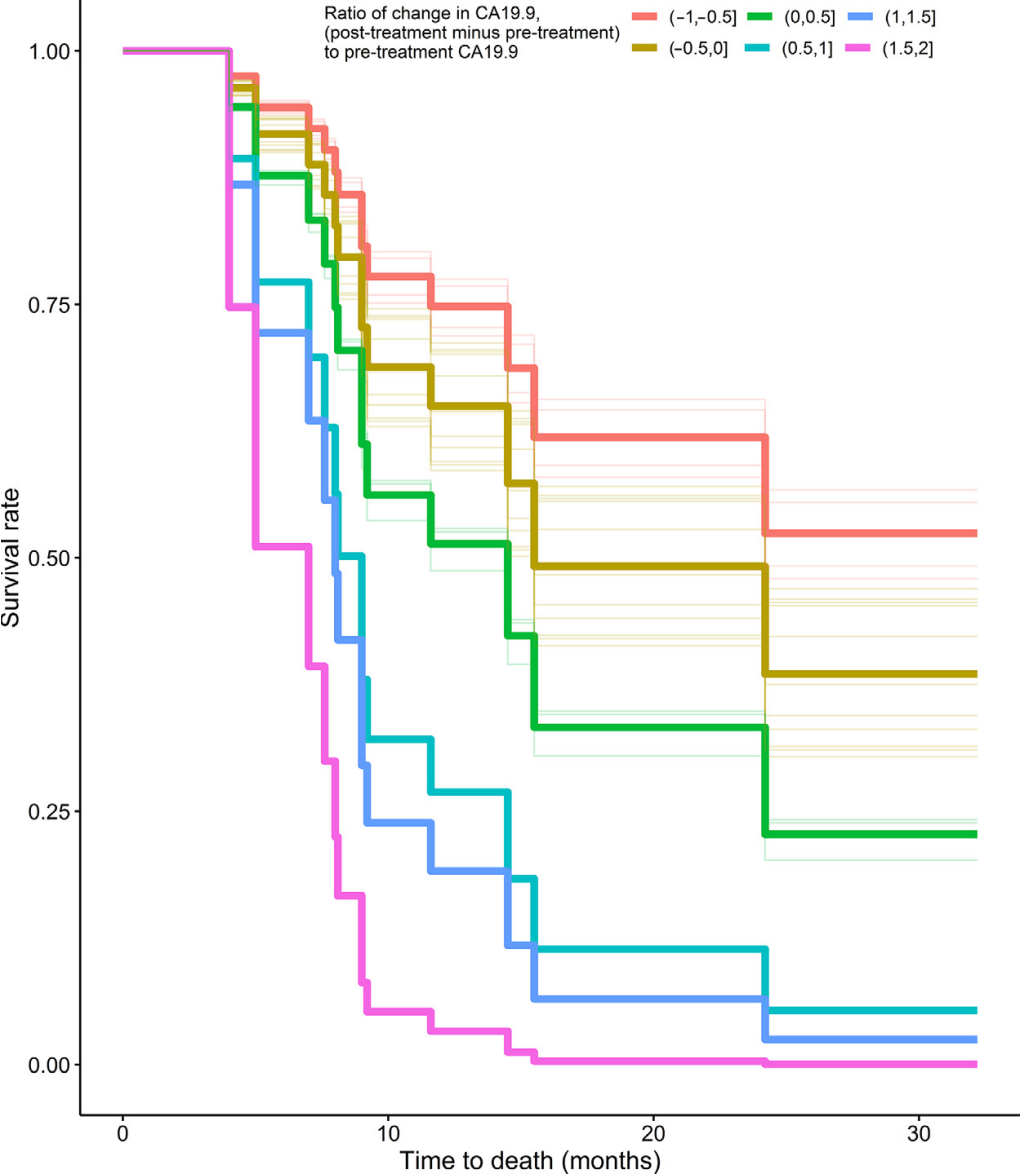
Adjusted survival curves based on magnitude of change in CA 19.9. Group 1 (−1, −0.5) is more than 50% decrease; Group 2 (−0.5, 0) is 0–50% decrease; Group 3 (0, 0.5) is 0–50% increase; Group 4 (0.5, 1) is from 50% to 100% increase; Group 5 (1, 1.5) is 100–150% increase; Group 6 (1.5, 2) is from 150% to 200% increase.

The only factor that was found to have a statistical significant effect on survival was the change in CA19‐9 values before and after treatment. As expected a decrease indicated longer survival and an increase was related to shorter survival. It is likely that there are further factors that may be of significance which were not detected due to the small number of patients in our study. We further looked at the magnitude of change in CA19‐9 and found that a decrease of 10% from the initial CA19‐9 value reduced the monthly hazard rate of death by 9.7% (95% CI: 4–15.7%). Figure 3 illustrates the estimated survival curve for specific group of patients defined by the magnitude of decrease or increase in CA19‐9. Further research is needed to validate if indeed the magnitude of change in CA19‐9 in pancreatic SBRT is a reliable prognostic predictor.

We acknowledge this paper's limitation on the ability for robust comparisons due to the small patient number and heterogeneity. But with these encouraging preliminary results and the apparent benefits seen, pancreatic SBRT remains an exciting field for further investigation. It must be explored in a randomised setting to validate the use of this novel technique and include studies focusing on organ tolerance, combining systemic and/or immunotherapy, molecular profiling, biochemical prognostic factors and robust quality of life measurements.

## Conclusion

Our institute's experience in SBRT for locally advanced unresectable pancreatic cancer has demonstrated promising local control rates which are higher than conventional external beam radiation therapy regimens, with minimal toxicities. In view of these findings, we find this treatment approach a feasible option for selected patients. Our results are in keeping with other centres internationally who have explored the role of SBRT in the management of locally advanced pancreatic cancer. To our knowledge, this is the first published data in the Oceanic region looking into this. Further phase three studies will likely validate the use of SBRT as standard treatment for locally advanced unresectable pancreatic cancer.

## Conflict of Interest

The authors declare no conflict of interest.

## References

[1] Australian Institute of Health and Welfare . ACIM (Australian Cancer Incidence and Mortality) Books: Pancreatic cancer. AIHW, Canberra, 2017.

[2] Cameron JL , Crist DW , Sitzmann JV , et al. Factors influencing survival after pancreaticoduodenectomy for pancreatic cancer. Am J Surg 1991; 161: 120–4. 1987845 10.1016/0002-9610(91)90371-j

[3] Wilkowski R , Wolf M , Heinemann V . Primary advanced unresectable pancreatic cancer. Recent Results Cancer Res 2008; 177: 79–93. 18084950 10.1007/978-3-540-71279-4_10

[4] Hammel P , Huguet F , van Laethem JL , et al. Effect of chemoradiotherapy vs chemotherapy on survival in patients with locally advance pancreatic cancer controlled after 4 months of gemcitabine with or without erlotinib: the LAP07 randomized clinical trial. JAMA 2016; 315: 1844–53. 27139057 10.1001/jama.2016.4324

[5] Iacobuzio‐Donahue CA , Fu B , Yachida S , et al. DPC4 gene status correlates with patterns of failure in patients with pancreatic cancer. J Clin Oncol 2009; 27: 1806–13. 19273710 10.1200/JCO.2008.17.7188 PMC2668706

[6] Timmerman RD . An overview of hypofractionation and introduction to the issue of seminars in radiation oncology. Semin Radiat Oncol 2008; 18: 215–22. 18725106 10.1016/j.semradonc.2008.04.001

[7] Gastrointestinal Tumor Study Group . Treatment of locally unresectable carcinoma of the pancreas: Comparison of combined‐modality therapy (chemotherapy plus radiotherapy) to chemotherapy alone. J Natl Cancer Inst 1988; 80: 751–5. 2898536

[8] Loehrer PJ Sr , Feng Y , Cardenes H , et al. Gemcitabine alone versus gemcitabine plus radiotherapy with locally advance pancreatic cancer: An Eastern Cooperative Oncology Group trial. J Clin Oncol 2011; 29: 4105–12. 21969502 10.1200/JCO.2011.34.8904 PMC3525836

[9] Chauffert B , Mornex F , Bonnetain F , et al. Phase III trial comparing intensive induction chemoradiotherapy (60 Gy, infusional 5‐FU and intermittent cisplatin) followed by maintenance gemcitabine with gemcitabine alone for locally advanced unresectable pancreatic cancer. Definitive results of the 2000‐01 FFCD/SFRO study. Ann Oncol 2008; 19: 1592–9. 18467316 10.1093/annonc/mdn281

[10] Koong AC , Christofferson E , Le QT , et al. Phase II study to assess the efficacy of conventionally fractionated radiotherapy followed by a stereotactic radiosurgery boost in patients with locally advanced pancreatic cancer. Int J Radiat Oncol Biol Phys 2005; 63: 320–3. 16168826 10.1016/j.ijrobp.2005.07.002

[11] Didolkar MS , Coleman CW , Brenner MJ , et al. Image‐guided stereotactic radiosurgery for locally advanced pancreatic adenocarcinoma results of first 85 patients. J Gastrointest Surg 2010; 14: 1547–59. 20839073 10.1007/s11605-010-1323-7

[12] Polistina F , Constantin G , Casamassima F , et al. Unresectable locally advanced pancreatic cancer: A multimodal treatment using neoadjuvant chemoradiotherapy (gemcitabine plus stereotactic radiosurgery) and subsequent surgical exploration. Ann Surg Oncol 2010; 17: 2092–101. 20224860 10.1245/s10434-010-1019-y

[13] Mahadevan A , Jain S , Goldstein M , et al. Stereotactic body radiotherapy and gemcitabine for locally advanced pancreatic cancer. Int J Radiat Oncol Biol Phys 2010; 78: 735–42. 20171803 10.1016/j.ijrobp.2009.08.046

[14] Schellenberg D , Kim J , Christman‐Skieller C , et al. Single‐fraction stereotactic body radiation therapy and sequential gemcitabine for the treatment of locally advanced pancreatic cancer. Int J Radiat Oncol Biol Phys 2011; 81: 181–8. 21549517 10.1016/j.ijrobp.2010.05.006

[15] Goyal K , Einstein D , Ibarra RA , et al. Stereotactic body radiation therapy for nonresectable tumours of the pancreas. J Surg Res 2012; 174: 319–25. 21937061 10.1016/j.jss.2011.07.044 PMC4196857

[16] Lominska CE , Unger K , Nasr NM , et al. Stereotactic body radiation therapy for reirradiation of localized adenocarcinoma of the pancreas. Radiat Oncol 2012; 7: 74. 22607687 10.1186/1748-717X-7-74 PMC3441204

[17] Chuong MD , Springett GM , Freilich JM , et al. Stereotactic body radiation therapy for locally advanced and borderline resectable pancreatic cancer is effective and well tolerated. Int J Radiat Oncol Biol Phys 2013; 86: 516–22. 23562768 10.1016/j.ijrobp.2013.02.022

[18] Gurka MK , Kim C , He AR , et al. Stereotactic body radiation therapy (SBRT) combined with chemotherapy for unresected pancreatic adenocarcinoma. Am J Clin Oncol 2017; 40: 152–7. 25171298 10.1097/COC.0000000000000118 PMC4418949

[19] Moningi S , Dholakia AS , Raman SP , et al. The role of stereotactic body radiation therapy for pancreatic cancer. A single‐institution experience. Ann Surg Oncol 2015; 22: 2352–8. 25564157 10.1245/s10434-014-4274-5 PMC4459890

[20] Herman JM , Chang DT , Goodman KA , et al. Phase 2 multi‐institutional trial evaluating gemcitabine and stereotactic body radiotherapy for patients with locally advanced unresectable pancreatic adenocarcinoma. Cancer 2015; 121: 1128–37. 25538019 10.1002/cncr.29161 PMC4368473

[21] Lin JC , Jen YM , Li MH , et al. Comparing outcomes of stereotactic body radiotherapy with intensity‐modulated radiotherapy for patients with locally advanced unresectable pancreatic cancer. Eur J Gastroenterol Hepatol 2015; 27: 259–64. 25629569 10.1097/MEG.0000000000000283

[22] Tozzi A , Comito T , Alongi F , et al. SBRT in unresectable advanced pancreatic cancer. Preliminary results of a mono‐institutional experience. Radiat Oncol 2013; 8: 148. 23799996 10.1186/1748-717X-8-148 PMC3707803

[23] Petrelli F , Comito T , Ghidini A , et al. Stereotactic body radiation therapy for locally advanced pancreatic cancer: A systemic review and pooled analysis of 19 trials. Int J Radiat Oncol Biol Phys 2017; 97: 313–22. 28068239 10.1016/j.ijrobp.2016.10.030

[24] Murphy JD , Adusumilli S , Griffith KA , et al. Full‐dose gemcitabine and concurrent radiotherapy for unresectable pancreatic cancer. Int J Radiat Oncol Biol Phys 2007; 68: 801–8. 17379445 10.1016/j.ijrobp.2006.12.053

[25] Small W Jr , Berlin J , Freedman GM , et al. Full‐dose gemcitabine with concurrent radiation therapy in patients with nonmetastatic pancreatic cancer: A multicenter phase II trial. J Clin Oncol 2008; 26: 942–97. 18281668 10.1200/JCO.2007.13.9014

[26] Shinchi H , Takao S , Noma H , et al. Length and quality of survival after external‐beam radiotherapy with concurrent continuous 5‐fluorouracil infusion for locally unresectable pancreatic cancer. Int J Radiat Oncol Biol Phys 2002; 53: 146–50. 12007953 10.1016/s0360-3016(01)02806-1

[27] Schellenberg D , Goodman KA , Lee F , et al. Gemcitabine chemotherapy and single‐fraction stereotactic body radiotherapy for locally advanced pancreatic cnacer. Int J Radiat Oncol Biol Phys 2008; 72: 678–86. 18395362 10.1016/j.ijrobp.2008.01.051

[28] Chang DT , Schellenberg D , Shen J , et al. Stereotactic radiotherapy for unresectable adenocarcinoma of the pancreas. Cancer 2009; 115: 665–72. 19117351 10.1002/cncr.24059

[29] Rwigema JC , Parikh SD , Heron DE , et al. Stereotactic body radiotherapy in the treatment of advanced adenocarcinoma of pancreas. Am J Clin Oncol 2011; 34: 63–9. 20308870 10.1097/COC.0b013e3181d270b4

[30] Bae SH , Kim MS , Cho CK , et al. Predictor of severe gastroduodenal toxicity after stereotactic body radiotherapy for abdominopelvic malignancies. Int J Radiat Oncol Biol Phys 2012; 84: 469–74. 10.1016/j.ijrobp.2012.06.005 23078899

